# Effect of aqueous and ambient atmospheric environments on plasmon-driven selective reduction reactions

**DOI:** 10.1038/srep10269

**Published:** 2015-06-01

**Authors:** Qianqian Ding, Maodu Chen, Yuanzuo Li, Mengtao Sun

**Affiliations:** 1 Key Laboratory of Materials Modification by Laser, Electron, and Ion Beams (Ministry of Education), School of Physics and Optoelectronic Technology, Dalian University of Technology, Dalian 116024, People’s Republic of China; 2 Beijing National Laboratory for Condensed Matter Physics, Institute of Physics, Chinese Academy of Sciences, Beijing 100190, People’s Republic of China; 3 College of Science, Northeast Forestry University, Harbin 150040, People’s Republic of China

## Abstract

We successfully realised plasmon-driven selective reduction reactions of 2-amino-5-nitrobenzenethiol (2A-5-NBT) to 3,3’-dimercapto-4,4’-diaminoazobenzene , an azobenzene derivative, using surface-enhanced Raman scattering (SERS) spectroscopy, and supported by the theoretical calculations. The SERS spectra demonstrated that two 5-nitro groups of 2A-5-NBTs were selectively reduced to the –N=N– chemical bond of 3,3’-dimercapto-4,4’-diaminoazobenzene, whereas the 2-amine group of 2A-5-NBT remained unchanged. Our experimental results revealed that aqueous environments were preferable to ambient atmospheric environments for this selective reduction reaction. The product is very stable in aqueous environments. However, in ambient atmosphere environments, the product is not stable and can revert back to 2A-5-NBT, where the –N=N– chemical bond can be broken by plasmon scissors. The plasmon-induced catalytic reactions in aqueous environments could be used for the efficient synthesis of aromatic azobenzene derivative compounds, which are valuable chemicals that are widely used in the chemical industry as dyes, food additives and drugs.

Surface plasmon resonance (SPR) is a collective oscillation of surface conduction electrons that is excited by incident light at specific photon energies. This phenomenon has been exploited in plasmon-driven chemical reactions^1^,^2^,^3^,^4^,^5^,^6^,^7^,^8^,^9^,^10^, which have attracted significant attention because of their high throughput and low energy requirements. Hot electrons with high kinetic energy have been generated from plasmon decay^11^,^12^. These hot electrons play a key role in plasmon-induced chemical reactions by providing the required electrons and energy for the reactions to proceed.

Recently, extensive studies have demonstrated that p-aminothiophenol (PATP) absorbed on metal nanostructures can be selectively transformed into a new azo species, p,p’-dimercaptoazobenzene (DMAB), by a plasmon-driven catalytic reaction^13^,^14^,^15^,^16^,^17^. In this oxidation reaction, two 4-amine (-NH_2_) groups of PATP are selectively coupled to a –N=N– chemical bond to form DMAB. A similar aromatic compound, 4-nitrothiophenol (4-NBT), can also be catalyzed into DMAB with the assistance of plasmons^18^,^19^,^20^,^21^. In this reduction reaction, two 4-nitro groups of 4-NBT are selectively reduced to an azo bond. The two aforementioned reactions are strongly affected by the nature of the substrate materials^22^, the irradiation wavelength and power^23^,^24^, the solution pH^25^ and the ambient atmosphere^17^ or aqueous enviroment^26^. However, the plasmon-enhanced catalysed reactions on metallic catalysts can be monitored and controlled by surface-enhanced Raman scattering (SERS)^14^,^21^ and tip-enhanced Raman spectroscopy (TERS)^27^.

2-amino-5-nitrobenzenethiol (2A-5-NBT, see Fig. 1a) is an interesting molecule that contains benzyl, amine, nitro and -SH groups. This molecule can be used to study the competition between plasmon-driven oxidation and reduction in different environments, e.g., aqueous or ambient atmospheric environments. These three different molecules, 2,2’-dimercapto-4,4’-dintroazobenzene, 3,3’-dimercapto-4,4’-diaminoazobenzene, and 2,3’-dimercapto-4-nitro-4-amino-azobenzene (see Fig. 1b–d) could be oxidized or reduced by SPR. This molecule can also be used to determine the most stable environment for plasmon-driven chemical reactions.

In this study, we used SERS spectroscopy in conjunction with theoretical calculations to investigate the plasmon-driven selective reduction reaction of 2A-5-NBT, which was chemically absorbed on a roughened Ag substrate and was dimerized to a new azobenzene derivative compound, 3,3’-dimercapto-4,4’-diaminoazobenzene. This selective reduction reaction proceeded via the nitro group of 2A-5-NBT, and thus, oxidation via the amine group of 2A-5-NBT did not occur. The product was very stable in aqueous environments. However, in ambient atmospheric environments, the product was not stable and reverted to 2A-5-NBT, where the resulting –N=N– chemical bond could be further dissociated by plasmon scissors.

Our findings provide a novel environmentally friendly synthetic method for forming azobenzene derivative compounds, which are important and valuable industrial dyes^28^ and have potential applications as molecular wires and switches in molecular electronics^29^,^30^,^31^.

## Results

### Characterisation of SERS-active substrates

**Figure 2:**
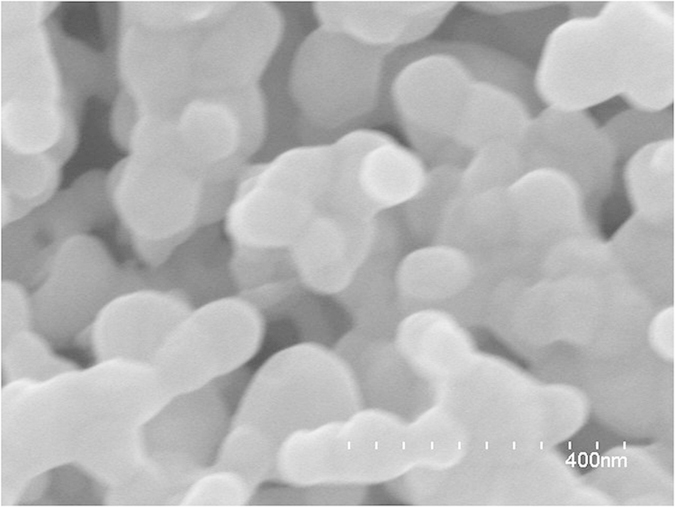
SEM image of Ag substrate.

The substrate for the SERS measurements was prepared using a previously reported method^26^. The SEM image (Fig. 2) of the roughened Ag electrode shows three-dimensional (3D) “hot spots” or 3D nanogaps^26^. The SEM image shows that the substrate was roughened along and perpendicular to the surface. This structure increased the number of “hot spots” and the intensity of the SPR for the plasmon-driven chemical reactions^32^.

### Plasmon-driven chemical reactions in an aqueous environment

**Figure 3:**
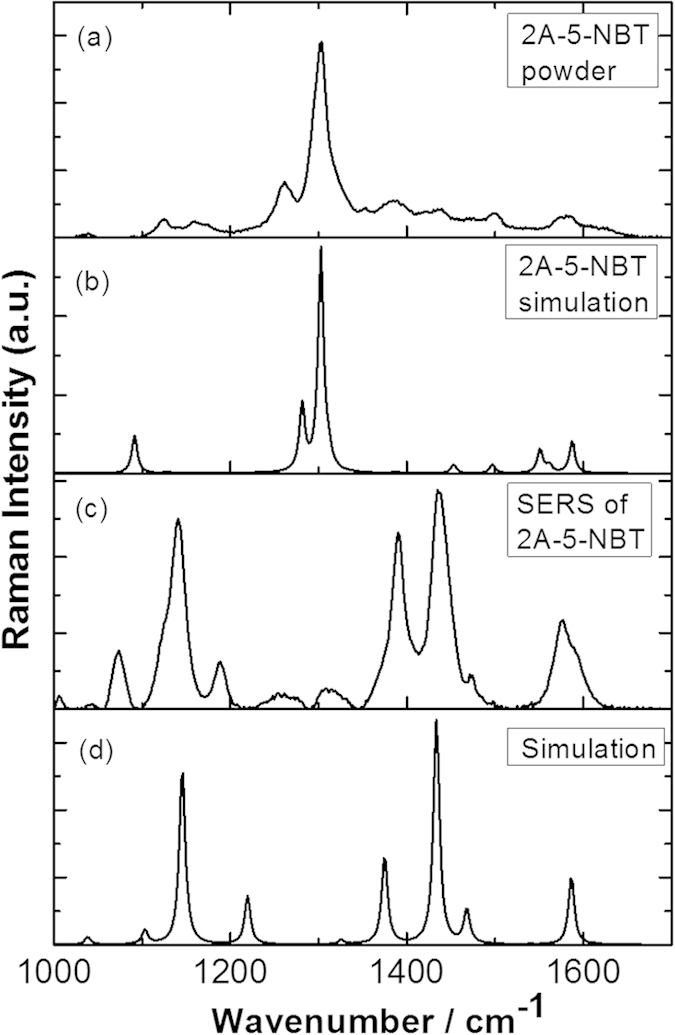
Molecular Raman spectra. (**a**) Raman spectrum of 2-amino-5-nitrobenzenethiol powder, (**b**) simulated Raman spectrum of 2-amino-5-nitrobenzenethiol, (**c**) SERS of 2-amino-5-nitrobenzenethiol in an aqueous environment and (**d**) simulated Raman spectrum of 3,3’-dimercapto-4,4’-diaminoazobenzene.

Figure 3(a) and (b) shows the measured Raman spectrum of the 2A-5-NBT powder and the corresponding simulated Raman spectrum, respectively, which are in good agreement. The theoretical calculations showed that the strongest Raman peak at 1302 cm^−1^ in Fig. 3(a) could be attributed to the -NO_2_ stretching mode of 2A-5-NBT.

**Figure 4:**
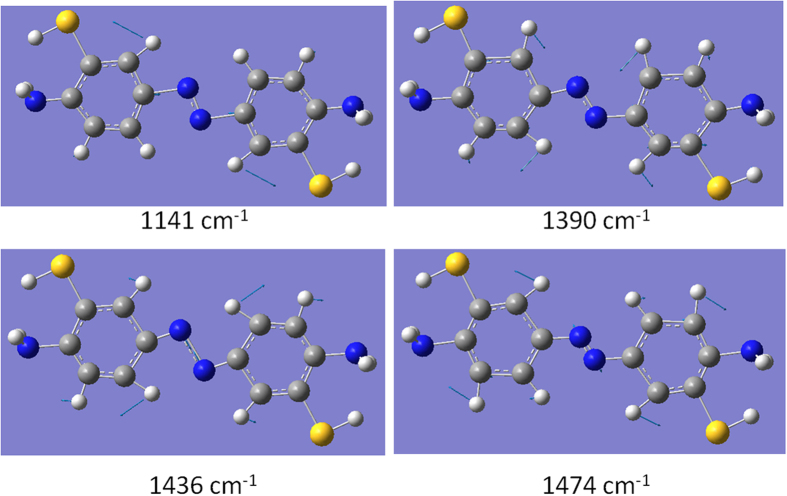
Molecular vibrational modes of 3,3’-dimercapto-4,4’-diaminoazobenzene.

Figure 3(c) shows the SERS spectrum of 2A-5-NBT that was measured in an aqueous environment. The profiles in Fig. 3(a) and (c) are significantly different from each other. SPR-assisted chemical reactions appear to have occurred for 2A-5-NBT in the aqueous environment. As discussed in the Introduction section, three types of molecules can be synthesised by different reduction or oxidation reactions. The simulated Raman spectrum for 3,3’-dimercapto-4,4’-diaminoazobenzene is shown in Fig. 3(d). When compared, Fig. 3(c) and (d) clearly shows that 2A-5-NBT was selectively reduced to 3,3’-dimercapto-4,4’-diaminoazobenzene via two nitro groups from 2A-5-NBT molecules, whereas the 2-amine group of 2A-5-NBT was unchanged. The vibrational modes of the strong Raman peaks of 3,3’-dimercapto-4,4’-diaminoazobenzene are shown in Fig. 4, where the peak at 1436 cm^−1^ was attributed to the –N=N– stretching mode.

**Figure 5:**
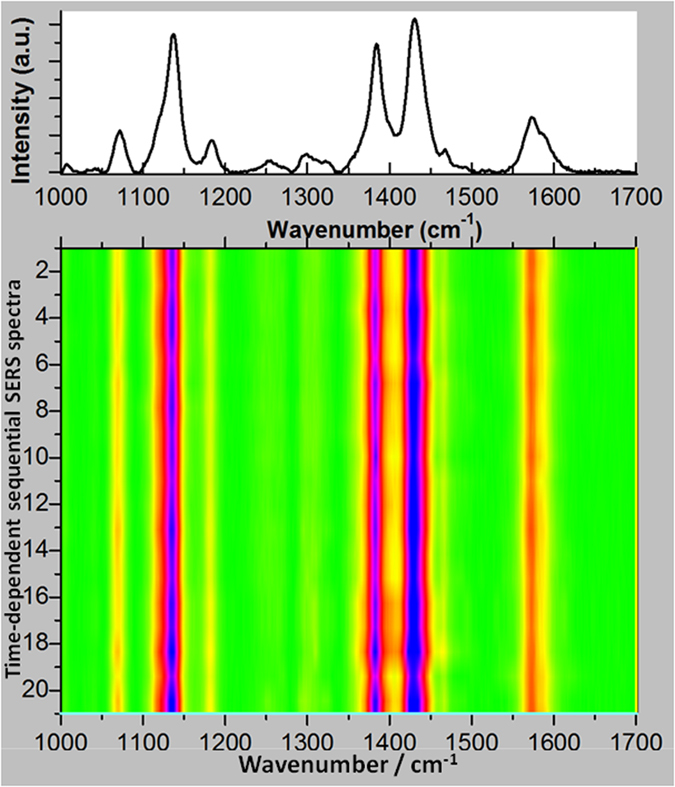
Time-dependent sequential SERS spectra in an aqueous environment. The acquisition time of each spectrum is 90 seconds, and the time interval is 3 minutes.

We studied the stability of plasmon-driven selective reductive reactions by measuring the time-dependent sequential SERS spectra in an aqueous environment (see Fig. 5). These plasmon-driven selective reduction catalysis reactions were found to be very efficient and the resulting product was very stable.

### Plasmon-driven chemical reactions in an ambient atmosphere environment

**Figure 6:**
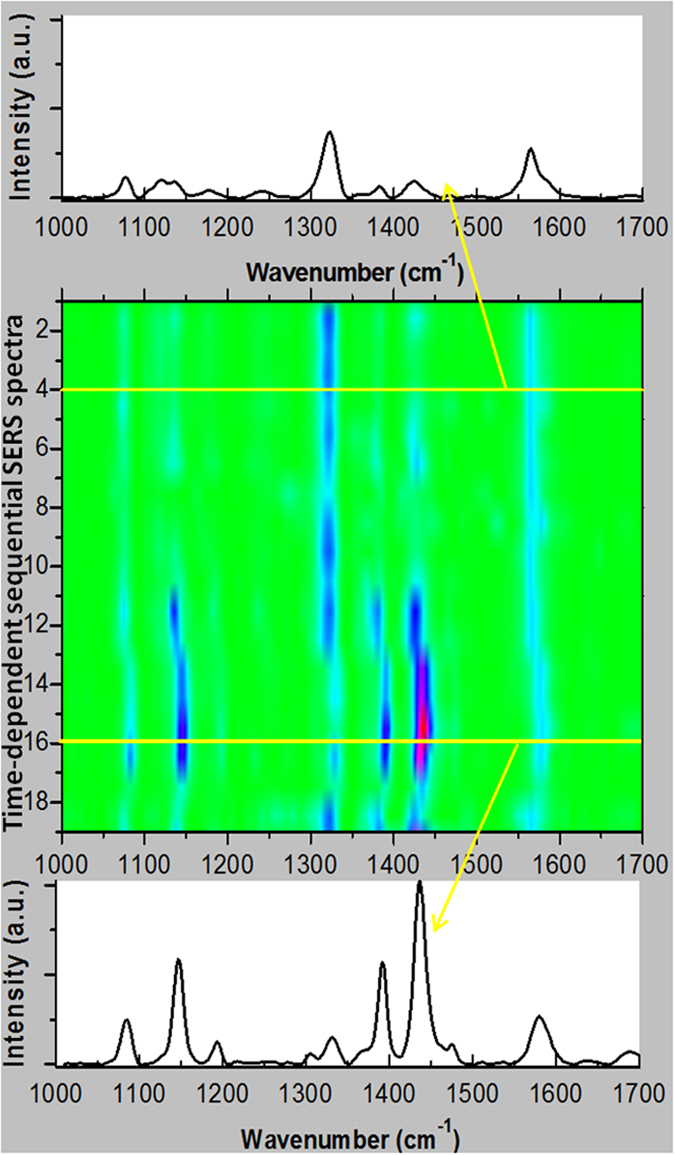
Time-dependent sequential SERS spectra in an ambient atmospheric environment. The acquisition time of each spectrum is 90 seconds, and the time interval is 3 minutes.

We studied the effect of different environments on plasmon-driven selectively catalysed reactions by measuring the time-dependent sequential SERS spectra (see Fig. 6). At the initial stages of the chemical reaction, the reactant 2A-5-NBT was clearly observed (see the upper spectrum in Fig. 6). With increasing time, 2A-5-NBT was selectively catalysed to 3,3’-dimercapto-4,4’-diaminoazobenzene (see the lower spectrum in Fig. 6). Subsequent SERS measurements showed that the product reverted to the reactant via the dissociation of the –N=N– chemical bond by plasmon scissors. There have been several reports on the dissociation of the –N=N– chemical bond by plasmon scissors using SERS^33^ and TERS^34^.

## Discussion

**Figure 7:**
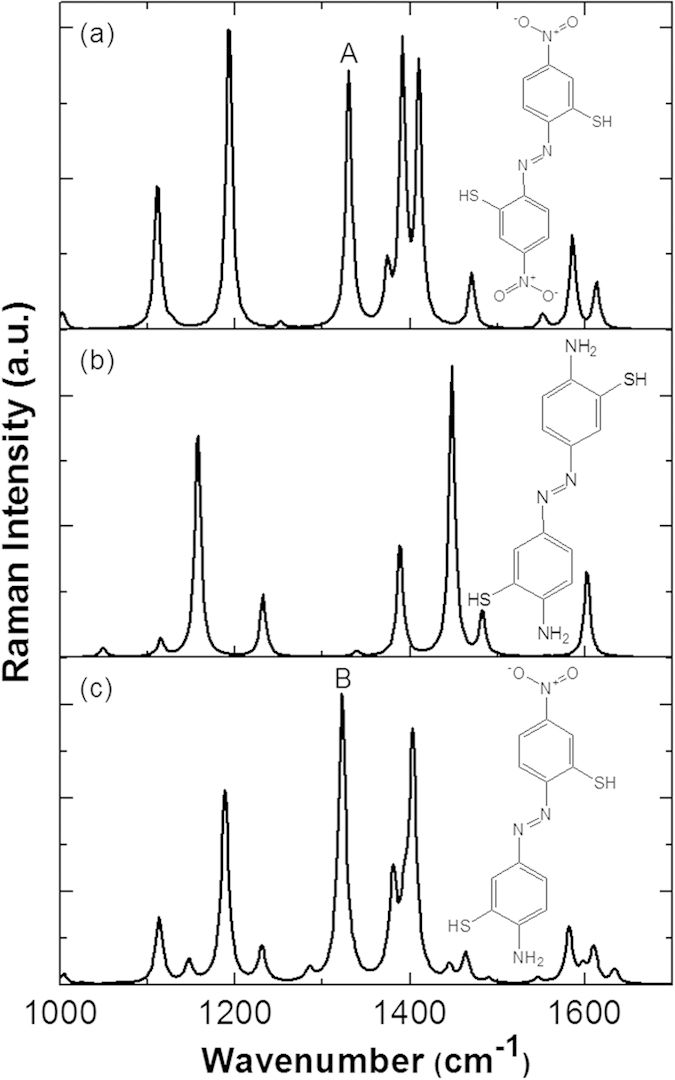
Simulated Raman spectra. (**a**) 2,2’-dimercapto-4,4’-dintroazobenzene, (**b**) 3,3’-dimercapto-4,4’-diaminoazobenzene, and (**c**) 2,3’-dimercapto-4-nitro-4-amino-azobenzene.

2A-5-NBT contains an amine group (–NH_2_) and a nitro group (–NO_2_); hence, it is difficult to ascertain which nitrogenous group participates in the selective formation of the azo bond. We considered three potential coupling modes in simulating the Raman spectra of the corresponding products 2,2’-dimercapto- 4,4’-dintroazobenzene, 3,3’-dimercapto-4,4’-diaminoazobenzene, and 2,3’-dimercapto-4-nitro-4-amino-azobenzene (see Fig. 7(a)–(c)), which are denoted as molecules A, B and C, respectively. The molecular structure graph of molecule A in Fig. 7(a) shows that a –N=N– chemical bond could form at the 2’2 coupling via two 2-amine groups from 2A-5-NBT molecules. Similarly, a second possible molecule B (see Fig. 7(b)) could be selectively produced through the coupling of two 5-nitro groups of 2A-5-NBT molecules. The plasmon could also convert the 2A-5-NBT to a third possible product, C, (see Fig. 7(c)) by the selective coupling of a 2-amine group and a 5-nitro group of 2A-5-NBT.

**Figure 8:**
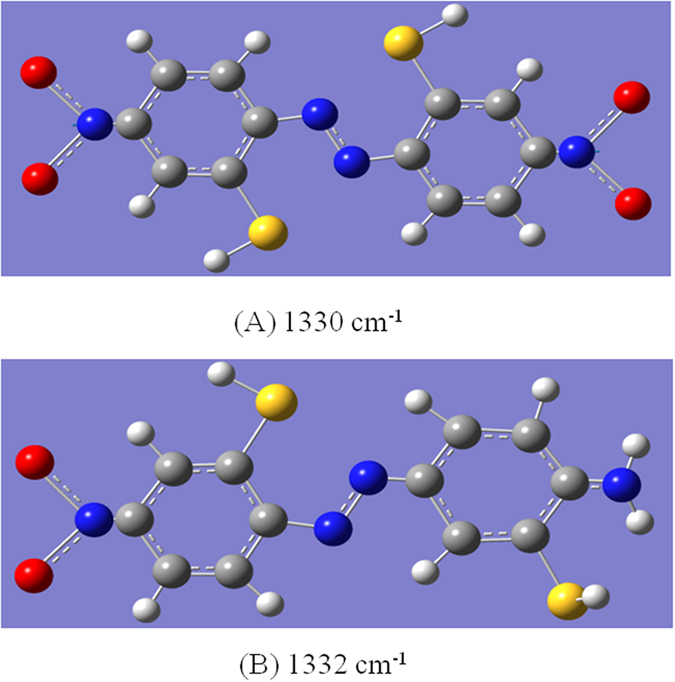
Molecular vibrational modes. (**a**) -NO_2_ vibrational modes of 2,2’-dimercapto- 4,4’-dintroazobenzene, and (**b**) -NO_2_ vibrational modes of 2,3’-dimercapto-4-nitro-4-amino-azobenzene.

We determined the selected product from the plasmon-driven catalytic coupling reaction by comparing the SERS spectrum of 2A-5-NBT on the roughened Ag substrate with the calculated Raman spectra for the three products. For molecules A and C, the strong Raman peaks at approximately 1330 cm^−1^ were attributed to the NO_2_ stretching modes (see Fig. 8) that were not observed in the experimental results in Figs 3(c) and 5, which is a distinct and unique experimental signature. The simulated Raman spectrum in Fig. 7b was consistent with the experimental SERS spectrum in Fig. 3(c). Thus, we concluded that 2A-5-NBT was selectively reduced to 3,3’-dimercapto-4,4’-diaminoazobenzene. The reduction reaction occurred before the oxidation of the molecules with amine and nitro groups. Different environments were found to strongly influence the stability, probability and efficiency of selective catalysis reactions by plasmons.

## Methods

2A-5-NBT was purchased from Beijing Kaida Co., according to the customer’s requirements. The NMR spectrum of 2A-5-NBT is presented in the supporting information.

The Ag substrate (a single-crystal silver rod of 99.99% purity) was polished with emery paper and cleaned with Milli-Q water in an ultrasonic bath. The Ag substrate was then placed in a typical electrochemical cell containing a 0.1 M Na_2_SO_4_ solution for roughening. A double potential step was used to roughen the surface by applying a voltage of +0.25 V for 8 s and then applying a voltage of −0.35 V.

The Raman spectrum of the 2A-5-NBT powder and the SERS spectrum of 2A-5-NBT (1 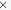
10^−6^ M) were measured using Raman spectroscopy (Renishaw inVia system) with a laser incident wavelength of 632.8 nm.

All of the theoretical calculations were performed with the Gaussian 09 suite using density functional theory^35^ and the PW91PW91 functional^36^ with b3lyp/6-311++g(d,p) for the reactant and the 6-31G(d) basis set for the three products.

## Additional Information

**How to cite this article**: Ding, Q. *et al*. Effect of aqueous and ambient atmospheric environments on plasmon-driven selective reduction reactions. *Sci. Rep.* **5**, 10269; doi: 10.1038/srep10269 (2015).

## Supplementary Material

Supplementary Information

## Figures and Tables

**Figure 1:**
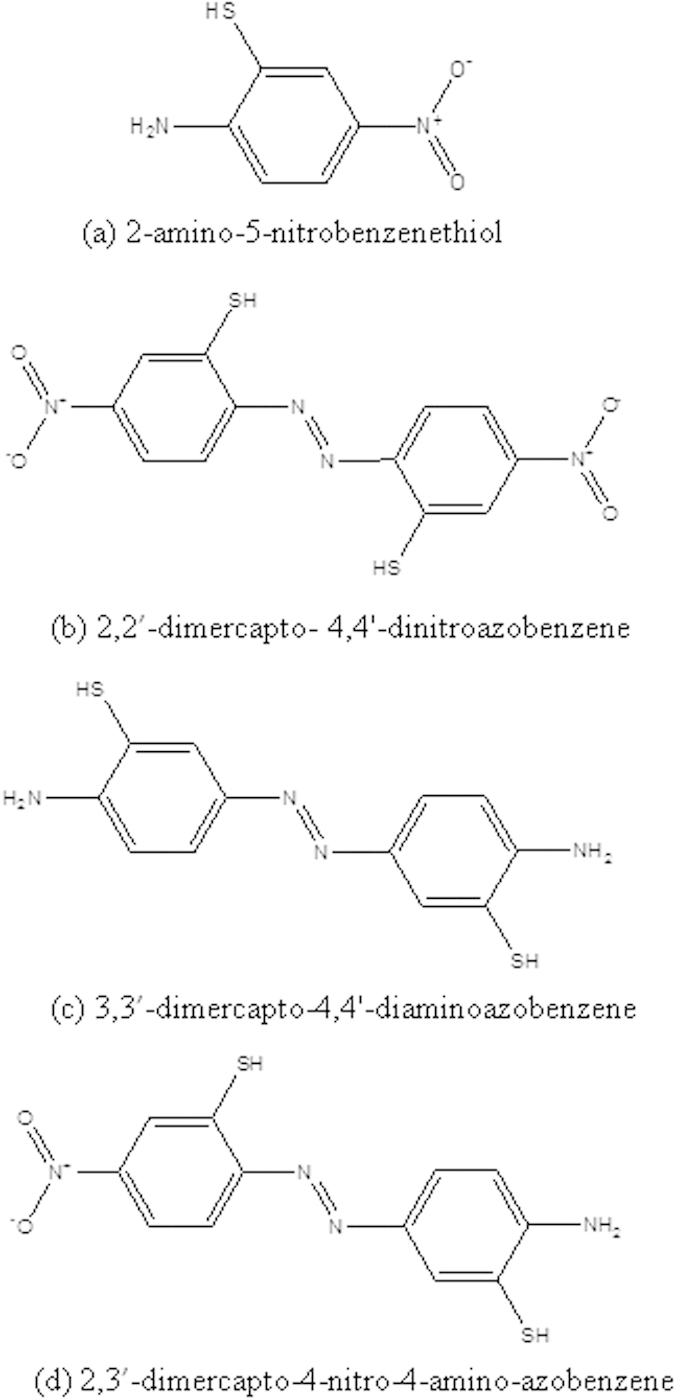
Molecular structures. (**a**) 2-amino-5-nitrobenzenethiol, (**b**) 2,2’-dimercapto- 4,4’-dintroazobenzene, (**c**) 3,3’-dimercapto-4,4’-diaminoazobenzene, and (**d**) 2,3’-dimercapto-4-nitro-4-amino-azobenzene.

## References

[b1] Chen C. J. & Osgood R. M. Near-field photochemical imaging of noble metal nanostructures. Phys. Rev. Lett. 50, 1705 (1983).

[b2] Hubert C. *et al.* Near-field photochemical imaging of noble metal nanostructures. Nano Lett. 5, 615–619 (2005). 15826096 10.1021/nl047956i

[b3] Tsuboi Y., Shimizu R., Shoji T. & Kitamura N. Near-infrared continuous-wave light driving a two-photon photochromic reaction with the assistance of localized surface plasmon. J. Am. Chem. Soc. 131, 12623–13267 (2009). 19681601 10.1021/ja9016655

[b4] Zhu H., Ke X., Yang X., Sarina S. & Liu H. Reduction of nitroaromatic compounds on supported gold nanoparticles by visible and ultraviolet light. *Angew. Chem. Int. Ed*. 49, 9657–9661 (2010). 10.1002/anie.201003908 21053223

[b5] Christopher P., Xin H. & Linic S. Visible-light-enhanced catalytic oxidation reactions on plasmonic silver nanostructures. Nature Chem. 3, 467–472 (2011). 21602862 10.1038/nchem.1032

[b6] Xie W., Herrmann C., Kompe K., Haase M. & Schlucker S. Synthesis of bifunctional Au/Pt/Au Core/Shell nanoraspberries for *in situ* SERS monitoring of Platinum-catalyzed reactions. J. Am. Chem. Soc. 133, 19302–19305 (2011). 22053855 10.1021/ja208298q

[b7] Navalon S., de Miguel M., Martin R., Alvaro M. & Garcia H. Enhancement of the catalytic activity of supported gold nanoparticles for the Fenton reaction by light. J. Am. Chem. Soc. 133, 2218–2226 (2011). 21280633 10.1021/ja108816p

[b8] Silva C. G., Juarez R., Marino T., Molinari R. & Garcıa H. Influence of excitation wavelength (UV or visible light) on the photocatalytic activity of titania containing gold nanoparticles for the generation of hydrogen or oxygen. J. Am. Chem. Soc. 133, 595–602 (2011). 21142160 10.1021/ja1086358

[b9] Wadayama T. & Yokawa M. Hot-electron assisted reaction of p-nitrobenzoic acid adsorbed on metal–insulator–metal tunnel junction’s electrode surface. Chem. Phys. *Lett*. 428, 348–351 (2006).

[b10] Lee J. *et al.* Plasmonicphotoanodes for solar water splitting with visible light. *Nano Lett*. 12, 5014–5019 (2012). 22916955 10.1021/nl302796f

[b11] Knight M., W., Sobhani H., Nordlander P. & Halas N. Photodetection with active optical antennas. Science 332, 702–704 (2011). 21551059 10.1126/science.1203056

[b12] Zhang Z. L. *et al.* Insights into the nature of plasmon-driven catalytic reactions by HV-TERS. Nanoscale. 5, 3249–3252 (2013). 23512070 10.1039/c3nr00352c

[b13] Wu D. Y., *et al.* Surface catalytic coupling reaction of p-Mercaptoaniline linking to silver nanostructures responsible for abnormal SERS enhancement: a DFT study. *J. Phys. Chem. C*. 113, 18212–18222 (2009).

[b14] Fang Y., Li Y., Xu H. X. & Sun M. T. Ascertaining p, p’-dimercaptoazobenzene produced from p-aminothiophenol by selective catalytic coupling reaction on silver nanoparticles. Langmuir. 26, 7737–7746 (2010). 20455558 10.1021/la904479q

[b15] Huang Y. F. *et al.* When the signal is not from the original molecule to be detected: chemical transformation of para-aminothiophenol on Ag during the SERS measurement. J. Am. Chem. Soc. 132, 9244–9246 (2010). 20527877 10.1021/ja101107z

[b16] Xu P. *et al.* Mechanistic understanding of surface plasmon assisted catalysis on a single particle: cyclic redox of 4-aminothiophenol. Sci. Rep. 3, 2997 (2013). 24141289 10.1038/srep02997 PMC3801115

[b17] Huang Y. F. *et al.* Activation of Oxygen Gas on Au and Ag Nanoparticles Assisted by Surface Plasmon Resonances. Angew. Chem., Int. Ed. 53, 2353–2357 (2014). 10.1002/anie.201310097 24481674

[b18] Dong B., Fang Y. R., Xia L. X., Xu H. X. & Sun M. T. Is 4-nitrobenzenethiol converted to p,p’-dimercaptoazobenzene or 4-aminothiophenol by surface photochemistry reaction? J. Raman Spectrosc. 42, 1205–1206 (2011).

[b19] Sun M. T. & Xu H. X. A novel application of plasmonics: plasmon driven surface catalyzed reactions. Small 8, 2777–2786 (2012). 22777813 10.1002/smll.201200572

[b20] van S. Lantman E. M., Deckert-Gaudig T., Mank A. J. G., Deckert V. &Weckhuysen B. M. Catalytic processes monitored at the nanoscale with tip enhanced Raman spectroscopy. Nature Nanotech. 7, 583–586 (2012). 10.1038/nnano.2012.131 22902959

[b21] Xie W., Walkenfort B. & Schlucker S. Label-Free SERS Monitoring of Chemical Reactions Catalyzed by Small Gold Nanoparticles Using 3D Plasmonic Superstructures. *J. Am. Chem. Soc*. 135, 1657–1660 (2013). 23186150 10.1021/ja309074a

[b22] Dong B. *et al.* Substrate-, wavelength-, and time-dependent plasmon-assisted surface catalysis reaction of 4-nitrobenzenethiol dimerizing to p,p’-dimercaptoazobenzene on Au, Ag and Cu films. Langmuir , 27, 10677–10682 (2011). 21819110 10.1021/la2018538

[b23] Kang L. L. *et al.* Laser wavelength- and power-dependent plasmon-driven chemical reactions monitored using single particle surface enhanced Raman spectroscopy. Chem. Comm. 49, 3389–3391 (2013). 23440353 10.1039/c3cc40732b

[b24] Wang H. *et al.* Plasmon-driven surface catalysis in hybridized plasmonic gap modes. Sci. Rep. 4, 7087 (2014). 25404139 10.1038/srep07087 PMC4235312

[b25] Ji W. *et al.* PH-response mechanism of p-aminobenzenethiol on Ag nanoparticles revealed by two-dimensional correlation surface enhanced Raman scattering spectroscopy. J. Phys. Chem. Lett. 3, 3204–3209 (2012). 10.1021/jz301428e 26296030

[b26] Zhang X. *et al.* Plasmon-driven sequential chemical reactions in an aqueous environment. Sci. Rep. 4, 5407 (2014). 24958029 10.1038/srep05407 PMC4067756

[b27] Sun M. T., Zhang Z. L., Zheng H. R. & Xu. H. X. In-situ plasmon-driven chemical reactions revealed by high vacuum tip-enhanced Raman spectroscopy. Sci. Rep. 2, 647 (2012). 22970339 10.1038/srep00647 PMC3438462

[b28] Venkataraman K. in The Chemistry of Synthetic Dyes. (Academic Press, London, 1970).

[b29] Nowak A. M. & McCreery R. L. *In situ* Raman spectroscopy of bias-induced structural changes in nitrozaobenzene molecular electronic junctions. *J. Am. Chem. Soc*. 126, 16621–16631 (2004). 15600368 10.1021/ja045763r

[b30] Vives G. & Tour J. M. *Acc. Chem. Res*. Synthesis of single-molecule nanocars. *Acc. Chem. Res*. 42, 473–487 (2009). 19245268 10.1021/ar8002317

[b31] McCreery R. L. Molecular electronic junctions. *Chem. Mater*. 16, 4477–4496 (2004).

[b32] Cui L. *et al.* Plasmon-driven dimerization via S-S chemical bond in an aqueous environment. Sci. Rep. 4, 7221 (2014). 25427897 10.1038/srep07221 PMC4245520

[b33] Kim K., Kim K. L. & Shin K. S. Photoreduction of 4,4′-Dimercaptoazobenzene on Ag Revealed by Raman Scattering Spectroscopy. Langmuir 29, 183–190 (2013). 23252520 10.1021/la304159c

[b34] Sun M. T. *et al.* Plasmonic Scissors for Molecular Design. Chem. Eur. J. 19, 14958–14962 (2013). 24038434 10.1002/chem.201302610

[b35] Parr R. G. & Yang W. Density-functional theory of atoms and molecules (Oxford Univ. Press, Oxford, 1989).

[b36] Perdew J. P., Burke K. & Wang Y. Jacob’s ladder of density functional approximations for the exchange-correlation energy. Phys. Rev. B 54, 16533 (1996).

