# Supplementary material for: Effect of aqueous and ambient atmospheric environments on plasmon-driven selective reduction reactions
Source: Sci Rep. 2015 Jun 1;5:10269. doi: 10.1038/srep10269 (PMC4450751; doi:10.1038/srep10269)

Effect of aqueous and ambient atmospheric environments on plasmon-driven selective reduction reactions

Qianqian Ding,[1,2] Maodu Chen,[1,*] Yuanzuo Li,[3] Mengtao Sun[2, *]

1. Key Laboratory of Materials Modification by Laser, Electron, and Ion Beams (Ministry of Education), School of Physics and Optoelectronic Technology, Dalian University of Technology, Dalian 116024, People's Republic of China.
2. Beijing National Laboratory for Condensed Matter Physics, Institute of Physics, Chinese Academy of Sciences, P.O. Box 603–146, Beijing 100190, People's Republic of China
3. College of Science, Northeast Forestry University, Harbin 150040, People's Republic of China

* Corresponding Authors. Email: mtsun@iphy.ac.cn (M. T. Sun) and mdchen@dlut.edu.cn (M. D. Chen).

Fig. S1 NMR spectrum of 2-amino-5-nitrobenzenethiol

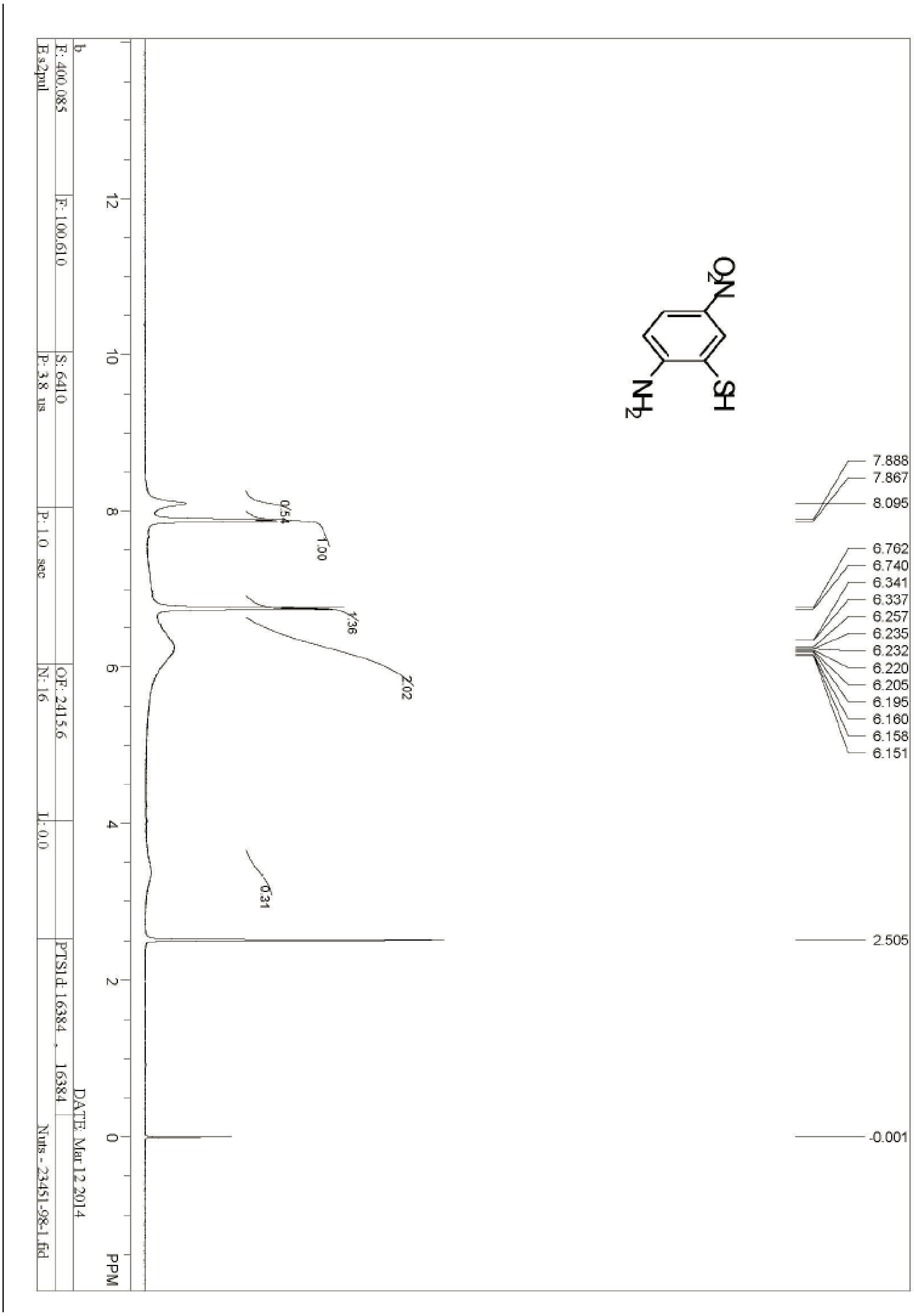

Supplement: Supplementary Information [file srep10269-s1.pdf]
